# The association between chronic pain and pre-and-post migration experiences in resettled humanitarian refugee women residing in Australia

**DOI:** 10.1186/s12889-022-13226-5

**Published:** 2022-05-07

**Authors:** Areni Altun, Sze-Ee Soh, Helen Brown, Grant Russell

**Affiliations:** 1 School of Public Health and Preventive Medicine, Monash University, Melbourne, Australia; 2 Department of General Practice, Monash University, Melbourne, Australia; 3 School of Exercise and Nutrition Sciences, Deakin University, Melbourne, Australia

**Keywords:** Chronic pain, Refugee health, Humanitarian, Resettlement

## Abstract

**Background:**

Refugee women are potentially at increased risk for chronic pain due to circumstances both in the pre-migration and post-settlement setting. However, this relationship between refugee-related challenges introduced along their migration trajectories and chronic pain remains unclear. This study will therefore examine the association between pre- and post-migration factors and chronic pain in refugee women five years into resettlement in Australia.

**Methods:**

The first five waves of data from the ‘Building a New Life in Australia’ longitudinal study of humanitarian refugees living in Australia was analysed using logistic regression models to investigate the association between predictor variables and chronic pain. The study outcome was chronic pain and predictors were migration process and resettlement factors in both the pre-and post-settlement setting.

**Results:**

Chronic pain was reported in 45% (*n* = 139) of women, and among these a further 66% (*n* = 120) also reported having a long-term disability or health condition that had lasted 12 months. Pre- migration factors such as increasing age (OR 1.08; 95% CI 1.05, 1.11) and women who migrated under the Women at Risk Visa category (OR 2.40; 95% CI 1.26, 4.56) had greater odds of experiencing chronic pain. Interestingly, post migration factors such as women with better general health (OR 0.04; 95% CI 0.01, 0.11) or those who settled within metropolitan cities (OR 0.29; 95% CI 0.13, 0.68) had lower odds of experiencing chronic pain, and those who experience discrimination (OR 11.23; 95% CI 1.76, 71.51) had greater odds of experiencing chronic pain.

**Conclusion:**

Our results show that there is a high prevalence of chronic pain in refugee women across the initial years of resettlement in Australia. This may be in part due to pre-migration factors such as age and migration pathway, but more significantly the post migration context that these women settle into such as rurality of settlement, poorer general health and perceived discriminatory experiences. These findings suggest that there may be many unmet health needs which are compounded by the challenges of resettlement in a new society, highlighting the need for increased clinical awareness to help inform refugee health care and settlement service providers managing chronic pain.

**Supplementary Information:**

The online version contains supplementary material available at 10.1186/s12889-022-13226-5.

## Background

By the end of 2020 82.4 million people were forcibly displaced worldwide as a result of conflict, persecution or human rights violations and of these, 26 million were refugees [1]. Approximately 85% of refugees are hosted in developing countries, however the remainder settle in countries such as Australia, which offer resettlement to 18,750 refugees with humanitarian needs each year [1, 2]. Whilst the experience of migration has been shown to contribute to adverse effects on overall health, education and livelihood, it may also continue to have longer term impacts on these factors, particularly on chronic pain [3].

Worldwide, the burden of chronic pain is escalating and has rapidly become the leading cause of long-term disability [4]. Pain is regarded as chronic when it lasts or recurs for more than three months [5]. Although there may be a single precipitating event in the genesis of chronic pain, such as an injury, there remains a series of factors that affect the duration, intensity and consequences of chronic pain [6]. Population based studies show that the prevalence of chronic pain is inversely related to socio-economic factors [4] with evidence that people who are socioeconomically deprived, who experience low levels of education, perceived income inequalities, and high levels of neighbourhood unrest are not only more likely to experience chronic pain, but also exhibit greater symptom severity and pain-related disability [7, 8]. Furthermore, women are more likely to experience pain and adopt poorer coping strategies leading to greater pain intensity and higher pain-related disability than men [4]. Pain closely interacts with social power structures, which means that marginalised groups, particularly women, are both more likely to experience pain and also more likely to have it regarded with doubt and inadequate care [9, 10].

Refugee women are one of the most vulnerable groups in our society and report some of the highest rates of chronic pain [11]. Whilst people from a refugee background may experience numerous vulnerabilities such as gender inequality, poverty, and social trauma [3], refugee women are at an increased risk to these vulnerabilities at times of armed conflict, humanitarian crisis, and displacement. Many women who migrate to Australia have been subjected to multiple traumas and as a result, chronic pain is a frequently exhibited health condition, affecting between 66 to 98 percent of traumatised people [12], often endured with scepticism and stigma from others [13]. Furthermore, the time following migration is recognised as a time of crisis, stress and adjustment and resettlement concerns such as housing, employment, and financial stress, can create greater psychological distress compounding the chronic pain experience. For treatment plans and prevention strategies to be effective in women of a refugee background, chronic pain needs to be understood in the context of broader social, biological, psychological and physical settings to ensure all individuals are able to use and engage with the appropriate health services [4].

There are substantial and complex ethnic variations in the prevalence and consequences of chronic pain, although the mechanisms behind these remain poorly understood. To date, much of the research investigating chronic pain in refugee people is not gender specific nor using longitudinal datasets [14, 15]. This has meant that information is not available on the full spectrum of chronic pain in refugee women, but rather a brief comparison and interpretation of a single moment of the resettlement experience. Longitudinal approaches that collect data on pain across several years may overcome this knowledge gap, where refugee women’s journey with chronic pain can be better explored. Understanding the migratory experience both before and after resettlement can provide health care providers with valuable insight into the needs of refugee women who are living with chronic pain.

The Building a New Life in Australia (BNLA) study is a five-year longitudinal, population-level cohort study of recently arrived humanitarian migrants in Australia. Using data from the first five years of the BNLA longitudinal survey, we examined chronic pain in refugee women. Other analyses of the BNLA survey have been published [16–20], but none have investigated the impact of migration on chronic pain of the BNLA respondents. With evidence of increasing refugee populations in Australia, it is important for resettlement nations to understand the long-term health needs and settlement prospects of refugee women so that timely and appropriate support services can be provided [16, 21]. However, in order do to so, we need to understand the extent to which migration factors shape the long-term experience of chronic pain. This study therefore aimed to identify the association between chronic pain and pre-and-post migration experiences in resettled humanitarian refugee women residing in Australia.

## Methods

### Study design

We conducted a secondary analysis of the five waves of the BNLA study. The BNLA is a nationally representative, longitudinal cohort study examining the first five years (2013- 2018) of resettlement in a humanitarian refugee population [22]. The study was conducted by the Australian Government’s Institute of Family Studies which examined how humanitarian refugees settle into a new life in Australia [22]. The Australian Federal Government’s Department of Social Services funded the BNLA study and all potentially identifying details from survey responses were deemed confidential to maintain the anonymity of respondents. Further information about the BNLA study design can be found in publicly available documents [22]. Our secondary analysis used data from waves one, three and five and data collection for these waves occurred between October 2013 and March 2014 (wave one), October 2015 and February 2016 (wave three), and October 2017 and February 2018 (wave five).

### Study population and sampling

**Fig. 1:**
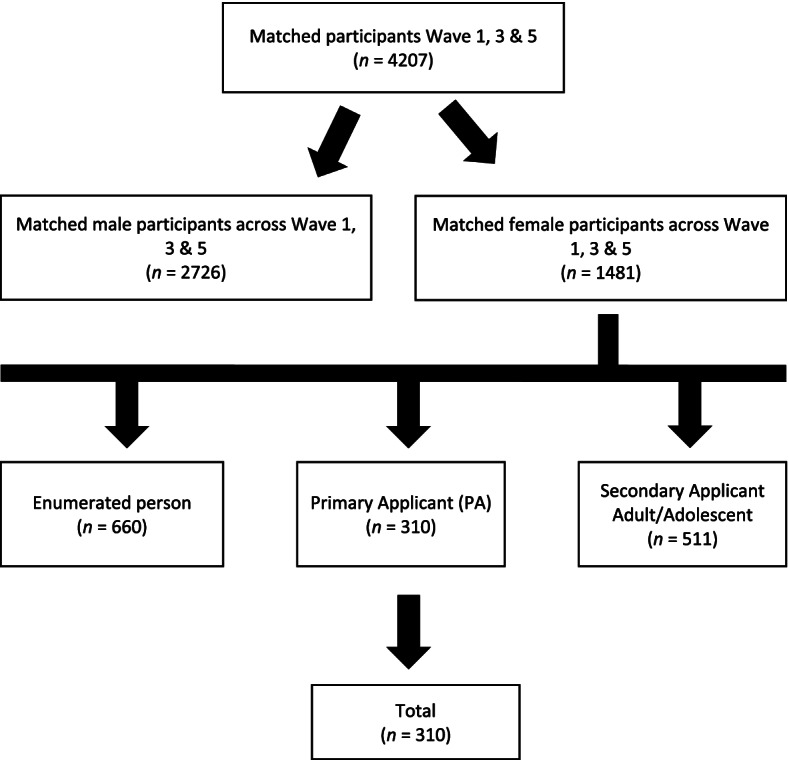
Flow chart illustrating participant eligibility

The BNLA cohort comprised of individuals aged 15 years and over who had been granted a permanent humanitarian visa by the Australian Government who first settled in Australia from May to October 2013 [22]. Eligible participants were identified via the Australian Department of Immigration and Border Protection settlement database from eleven locations around Australia to ensure valid spread of participant data nationally. Principal applicants (PA) are the primary adults listed on the visa application and were the initial individuals contacted for participation and were considered as the lead participants for the study. For the purpose of the current study, onshore and offshore humanitarian refugee women who were PAs 18 years and over and who participated in all three waves were included in the analysis. This sample was derived from women who responded to the chronic pain outcome variable at wave one, three and five. A flow chart detailing our sampling of eligible participants is illustrated in Fig. 1.

### Data collection

The five waves of ‘Building A New Life in Australia’ data were obtained from a written survey through home visits (Waves 1, 3 and 5) or telephone interviews (Wave 2 and 4) for data collection. The BNLA written survey was translated into 14 different languages and 19 languages were covered for the survey (with the aid of interpreters) [22].

### Migration factors

**Fig. 2:**
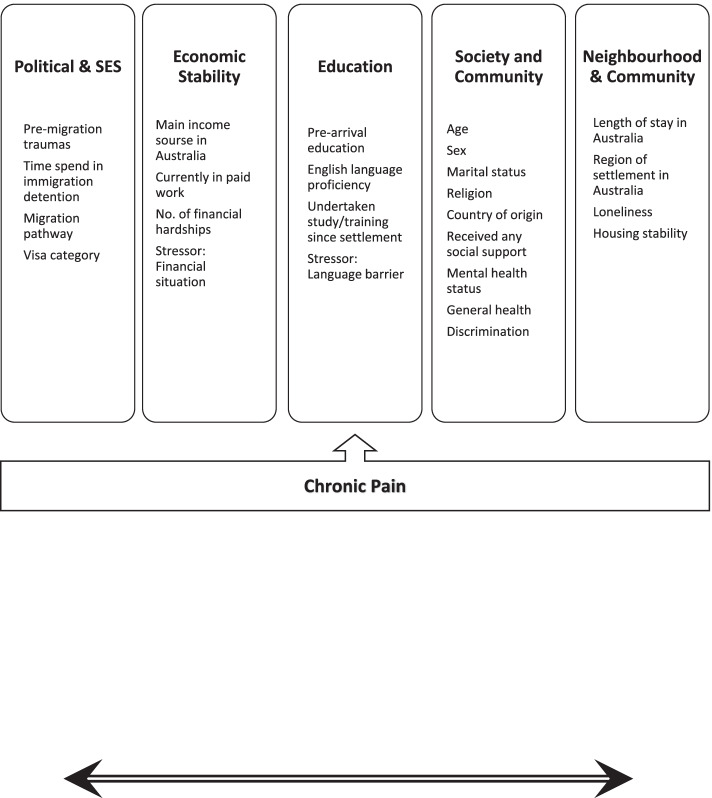
Chronic pain framework developed for analysis

Cross-Denny & Robinson’s social determinants of health model informed our variable selection from the BNLA survey that related to pre-and post-migration, resettlement, and health data. [23]. Cross-Denny & Robinson’s model uses five key areas that are particularly relevant for oppressed and marginalised populations. We added “Political, Socio-Economic” category to encompass the significant associations between structural/political factors (such as number of pre-migration traumas or migration pathway) and poorer general health among refugees. A review of the literature reporting predictors of refugee health outcomes informed the selection of predictor variables from the BNLA dataset to populate the social determinant of health model. Our adapted model includes five key determinants of refugee health and 25 predictor variables (See Fig. 2).

### Outcome variables

#### Chronic pain

The primary outcome for this study was self-reported chronic pain. To assess chronic pain in the current study, the question ‘How much bodily pain have you had during the past 4 weeks?’ was examined across waves one, three and five. Participants responded to this question using a six-point rating scale ranging from none (1), very mild (2), mild (3), moderate (4), severe (5) and very severe pain (6). For this study, we considered participants who responded with either no pain, very mild pain or mild pain as not having pain, while those who responded with moderate, severe or very severe as having pain [24]. Only participants who reported having pain across two consecutive waves (i.e. waves one and three, or waves three and five) were considered to have chronic pain as it reflects the presence of pain for 12 months or more.

#### Long-term disability

To determine whether women had a long-term disability in the current study, the question ‘Do you have a disability, injury or health condition that has lasted or is likely to last 12 months or more?’ was examined between waves one, three and five. Participants who responded ‘yes’ to this question at either wave one, three or five were considered to have a long-term disability.

### Statistical analysis

Data were analysed using Stata/IC 15.1 software [25]. Descriptive statistics were used to describe the overall characteristics of the sample population, such as socio-demographics, migration experience, and health outcomes. Logistic regression models were used to examine the association between the various migratory factors and chronic pain. Robust variance estimators were used to account for potential clustering [26].

A three-step modelling process was used to determine the pre-and post-migration factors associated with chronic pain. Firstly, univariate regressions were used to examine the association between predictor variables and chronic pain with variables retained at *p*$\le$ 0.1 [27]. Secondly, pre -and post-migration factors that had a moderate association with chronic pain were entered into two separate multivariate logistic regression models retained if *p*$\le$ 0.05. Collinearity was explored using the Variance Inflation Factor (VIF) and when collinearity was identified (VIF $\ge$ 2.5), the variable with the higher R^2^ on univariate analysis was retained for entry into the multivariate model.

A final model was computed using all statistically significant pre-and post-migration factors (*p*$\le$ 0.05) identified from the pre- and post – migration multivariate models. Interactions between predictor variables were also considered to identify any interaction effects. Lastly, we determined the proportion of women who report both chronic pain and long-term disability. This was used to inform our sub-group analysis which involved a logistic regression analysis to determine the pre-and post-migration factors associated with chronic pain in women who also reported having a long-term disability. Model fit was assessed using Akaike’s Information Criterion (AIC) [28].

### Ethics approval

The original BNLA study was approved by the Australian Institute of Family Studies ethics committee, which is registered with the National Health and Medical Research Council. Names of participants and potentially identifying information were withheld from the data source, meaning no individual can be identified by researchers. Ethics exemption for this secondary analysis of the data was granted by Monash University Human Research Ethics Committee.

### Role of the funding source

The BNLA is funded by the Australian Government’s Department of Social Services and the organisation was not involved in the preparation of this manuscript or the analyses reported.

## Results

### Demographics

At baseline, there were 310 women who were included in our secondary analysis of the BNLA study with a mean age of 41.3 years (SD = 12.9). Less than half were married or had a partner (*n* = 103; 33%) and the majority were born in North Africa or the Middle East (*n* = 166; 54%). Over a quarter had never attended school (*n* = 82; 26%) and only 9% (*n* = 28) of women held a university qualification. Before coming to Australia, 80% (*n* = 248) of women could speak English *“very well – well”.* A vast majority of the women had exposure to one or more traumatic experiences such as violence, imprisonment, conflict, extreme living conditions or other traumatic event (*n* = 272; 88%). Mostly, women had been in Australia less than six months (*n* = 279; 90%) and were living in metropolitan cities (*n* = 261; 84%). There was little financial stability with high unemployment (*n* = 308; 99%); high dependency on government income (*n* = 294; 95%), and at least one daily financial hardship (*n* = 138; 45%). The majority had stable housing with 48% (*n* = 148) reporting having a long-term lease or contract. Stress caused by not having work, language barriers, loneliness and discrimination was reported by 42% (*n* = 129), 68% (*n* = 212), 24% (*n* = 74), and 4% (*n* = 11) of participants, respectively. Over a quarter (*n* = 78; 25%) reported poor to very poor general health. See Table 1 for additional details on participant characteristics. A univariate regression analysis was conducted of each ethnic background against chronic pain, however no associations were found.

**Table 1:** Baseline characteristics of humanitarian refugee women in the Building A New Life in Australia project, 2013–14 (weighted data)

Characteristic	Description	Response	Total^a^ (*n* = 310)
**Pre-migration Factors**			
**Age, mean (years)**	Age	18 – 75 years	41.3 (12.9)
**Marital Status**	Married or has a Partner	Yes	103 (33.2%)
		No	207 (66.8%)
**Religion**	Religion	Buddhism	3 (0.97%)
		Christianity	135 (43.6%)
		Hinduism	3 (0.97%)
		Islam	134 (43.2%)
		Other	32 (10.3%)
		No Religion	3 (0.97%)
**Country of Birth**	Major groups based on the Standard Australian Classification of Countries major groups	North Africa and Middle East	166 (53.6%)
		South – East Asia	27 (8.7%)
		North – East Asia	1 (0.3%)
		Southern and Central Asia	108 (34.8%)
		Americas	1 (0.3%)
		Sub-Saharan Africa	7 (2.3%)
**Pre-arrival education**	Pre-arrival education	Never attended school	82 (26.4%)
		< 6 years of school	65 (21.0%)
		6 – 12 years of school	70 (22.6%)
		12 years + of school	50 (16.1%)
		Trade or Tech school	15 (4.8%)
		University Degree	28 (9.0%)
**Visa category**	Visa category	200 Refugee Visa	144 (46.4%)
		Onshore Protection/ Humanitarian Visa	35 (11.3%)
		204 Women at Risk Visa	131 (42.3%)
**Time spent in detention**	Time spent in detention	Yes	2 (0.7%)
		No	308 (99.3%)
**Number of pre-migration traumas**	Number of pre-migration traumas	None	38 (12.3%)
		1 – 2 traumas	169 (54.5%)
		3 or more traumas	103 (33.2%)
**Number of countries spent time in before coming to Australia**	Number of countries lived in between country of birth and Australia	None/not specified	50 (16.1%)
		1 Country	242 (78.1%)
		2 Countries	13 (4.2%)
		3 or more countries	5 (1.7%)
**Migration pathway**	Arrived in Australia via onshore^b^ or offshore migration pathway^c^	Onshore pathway	22 (7.1%)
		Offshore pathway	288 (92.9%)
**Post-migration Factors**			
**Region of settlement in Australia**	Region of settlement in Australia	Metropolitan cities	261 (84.2%)
		Regional Australia	49 (15.8%)
**Currently in paid employment**	Currently in paid employment in Australia	Yes	2 (0.7%)
		No	308 (99.3%)
**Main income source in Australia**	Main source of income in Australia	Government support	294 (94.8%)
		Non-Government support	16 (5.2%)
**No. daily financial hardships**	Number of daily financial hardships experienced in Australia	None	172 (55.5%)
		1 or more	138 (44.5%)
**Financial stressors**	Financial stressors	Yes	129 (41.6%)
		No	181 (58.4%)
**English speaking proficiency**	Currently understands spoken English	very well/well	248 (80.0%)
		not well/not at all	62 (20.0%)
**Undertaken further study/training in Australia**	Undertaken study or job training in Australia, other than English language classes	Yes	27 (8.7%)
		No	283 (91.3%)
**Stress-language barriers**	Language barriers as main source of stress in Australia	Yes	212 (68.4%)
		No	98 (31.6%)
**Housing stability**	Housing Stability	Temporary/Other	43 (13.9%)
		Short-term lease/contract	119 (38.4%)
		Long-term lease/contract	148 (47.7%)
**Mental Health Status PTSD8**	Meets intrusion, avoidance and hypervigilance criteria for PTSD	Yes	120 (38.7%)
		No	190 (61.3%)
**General Health**	General health status	Poor – Very poor	78 (25.2%)
		Good – Fair	170 (54.8%)
		Excellent – Very Good	62 (20.0%)
**Length of stay in Australia**	Length of stay in Australia	< 6 months	279 (90%)
		< 12 months	12 (3.9%)
		> 1 year	19 (6.1%)
**Stress-loneliness**	Loneliness as main source of stress in Australia	Yes	74 (23.9%)
		No	236 (76.1%)
**Received social support**	Received any religious, like ethnic or community support in Australia	Yes	148 (47.7%)
		No	162 (52.3%)
**Discrimination**	Discrimination as main source of stress in Australia	Yes	11 (3.6%)
		No	299 (96.4%)

^a^Data are provided as n (%) or mean (SD)

^b^Onshore pathway is available to those who wish to apply for asylum after arrival in Australia as an unauthorised maritime arrival or holder of valid visa (eg. tourist)

^c^Offshore pathway is available to those who may be eligible for resettlement to Australia, such as those identified by the UNHCR or those eligible for sponsorship to Australia

### Logistic regression results

#### Association between pre – migration factors and chronic pain

The univariate regression results can be found in Appendix A. The pre-migration variables that were moderately associated (*p*$\le$ 0.1) with chronic pain on univariate analysis were age, number of pre-migration traumas, marital status, pre-arrival education and migration pathway.

Table 2 provides the multivariate logistic regression results for the pre-migration factors, with chronic pain as the outcome variable and pre-migration factors as the predictor variables across the five years of follow-up. Our multivariate model showed that individual characteristics such as being older in age meant that the odds for having chronic pain were significantly higher (OR 1.08; 95% CI 1.05, 1.11) after controlling for other covariates. Furthermore, our results also showed that migration pathway was significantly associated with chronic pain (OR 2.40; 95% CI 1.26, 4.56). For example, women who arrived in Australia under the ‘Women at Risk (subclass 204)’ visa class had 2.4 times higher odds of reporting chronic pain compared to women who arrive in Australia under the Refugee (subclass 200) or Humanitarian visa (subclass 202) migration pathway. The Women at Risk Visa Subclass 204, is a visa in Australia which allows protection to the women who are living outside the country and who have been subjected to harassment, persecution, abuse or victimization on the basis of gender and do not have any male relative to protect them. We found no significant association between number of traumatic experiences, education level, or marital status in our multivariate model.

**Table 2:** Results from multivariate logistic regression analysis for pre-and-post migration factors using chronic pain and long-term disability as the outcome. OR-odds ratio; *p* = *p*-value; CI = confidence interval

Variable	Response	Chronic Pain			Long-term Disability		
		***OR***	***P***	***95% CI***	***OR***	***P***	***95% CI***
***PRE-MIGRATION FACTORS***							
**Age**	18 – 75 years	1.08	< 0.001	(1.05 – 1.11)	1.04	0.006	(1.01 – 1.08)
**No. of pre-migration traumas experienced**	None	-	-	-	-	-	-
	1 – 2	1.39	0.483	(0.56 – 3.45)	3.30	0.041	(1.05 – 10.34)
	3 or more	1.64	0.314	(0.63 – 4.28)	3.02	0.066	(0.93 – 9.85)
**Married or has a partner**	No	-	-	-	-	-	-
	Yes	0.96	0.907	(0.51 – 1.83)	1.01	0.982	(0.45 – 2.29)
**Pre-arrival Education**	Never attended school	-	-	-			-
	< 6 years of school	0.72	0.402	(0.34 – 1.55)	1.10	0.834	(0.44 – 2.77)
	6 – 12 years of school	0.55	0.127	(0.26 – 1.18)	1.25	0.664	(0.46 – 3.44)
	12 years + of school	0.56	0.166	(0.24 – 1.28)	0.88	0.791	(0.34 – 2.29)
	Trade or Tech school	0.86	0.786	(0.29 – 2.58)	1.72	0.460	(0.41 – 7.28)
	University Degree	0.52	0.188	(0.20 – 1.38)	1.32	0.711	(0.30 – 5.84)
**Visa Category**	200 Refugee Visa	-	-	-	-	-	-
	Onshore Protection/ Humanitarian Visa	1.36	0.464	(0.60 – 3.10)	1.06	0.904	(0.41 – 2.77)
	204 Women at Risk Visa	2.40	0.008	(1.26 – 4.56)	2.10	0.081	(0.91 – 4.83)
***POST-MIGRATION FACTORS***							
**Main Income Source in Australia**	Non- Government support	-	-	-	-	-	-
	Government support	5.32	0.070	(0.87 – 32.54)	6.48	0.155	(0.49 – 85.25)
**No. daily Financial Hardships**	None	-	-	-	-	-	-
	1 or more	1.24	0.458	(0.70 – 2.17)	1.26	0.537	(0.61 – 2.60)
**Stress – Financial**	No	-	-	-	-	-	-
	Yes	0.95	0.860	(0.53 – 1.71)	1.10	0.798	(0.52 – 2.37)
**English Speaking Proficiency**	Not well/not at all	-	-	-	-	-	-
	Very well/well	1.06	0.886	(0.48 – 2.37)	1.34	0.558	(0.50 – 3.62)
**Stress – Language barriers**	No	-	-	-	-	-	-
	Yes	1.31	0.458	(0.64 – 2.66)	1.00	0.991	(0.43 – 2.36)
**Mental Health Status PTSD8**	Does no meet criteria for PTSD	-	-	-	-	-	-
	Meets criteria for PTSD	1.65	0.091	(0.92 – 2.96)	1.80	0.175	(0.77 – 4.18)
**General Health**	Poor – Very poor	-	-	-	-	-	-
	Good – Fair	0.14	< 0.001	(0.07 – 0.28)	0.18	0.000	(0.07 – 0.45)
	Excellent – Very Good	0.04	< 0.001	(0.01 – 0.11)	0.04	0.000	(0.01 – 0.16)
**Region of Settlement in Australia**	Regional Australia	-	-	-	-	-	-
	Metropolitan cities	0.29	0.004	(0.13 – 0.68)	0.31	0.059	(0.09 – 1.04)
**Stress – Loneliness**	No	-	-	-	-	-	-
	Yes	1.70	0.136	(0.85 – 3.42)	1.50	0.364	(0.62 – 3.63)
**Stress – Discrimination**	No	-	-	-	-	-	-
	Yes	11.23	0.010	(1.76 – 71.51)	1.00	-	-

#### Association between post – migration factors and chronic pain

The post-migration variables that were moderately associated (*p*$\le$ 0.1) with chronic pain on univariate analysis were main income source, number of financial hardships, financial stressors, English language proficiency, language barrier, post-traumatic stress disorder (PTSD), general health, region of settlement in Australia, loneliness and discrimination (Appendix A).

Our multivariate analysis showed that post-migration factors such as region of settlement was significantly associated with chronic pain after controlling for other covariates (Table 2). Women who settled within metropolitan cities had lower odds of experiencing chronic pain compared to women who settled in more rural or remote regions (OR 0.29; 95% CI 0.13, 0.68). However, women who reported stress in the form of discrimination had higher odds of reporting chronic pain (OR 11.23; 95% CI 1.76, 71.51). Better general health was associated with lower odds of chronic pain. Women who reported ‘Very good – Excellent’ general health had lower odds of reporting chronic pain (OR 0.04; 95% CI 0.01, 0.11) which suggests that women who reported better general health had 96% times less odds of experiencing chronic pain. Our multivariate analysis showed that other post-migration variables such as main income source, financial hardship, financial stress, English speaking ability, PTSD history, or stress in the form of loneliness or language barriers were not significantly associated with chronic pain (Table 2). Interaction effects between post-migration factors were noted between region of settlement and general health. Women who settled in major cities had 20.5 times the odds of reporting better general health than those who settled in more regional parts of the country (OR 20.5, 95% 1.2, 338.8). Interactions effects between education levels and ethnicity were explored however no significant associations were found.

#### Association between pre-and post-migration factors and chronic pain

Our final model was informed by the previous two models. Age, visa class, region of settlement, discrimination and general health were all factors associated with chronic pain in the previous multivariate model and were therefore included in this final analysis. Age (OR 1.07; 95% CI 1.04, 1.09), Women at Risk visa class (OR 2.25, 95% CI 1.20, 4.24), better general health (OR 0.06; 95% CI 0.02, 0.16) and stress from discrimination (OR 13.78; 95% CI 3.15, 60.21) all continued to demonstrate a significant association with chronic pain in refugee women. Table 3 provides the multivariate logistic regression results for the final model involving pre-and-post migration factors, with chronic pain as the outcome variable and pre- and-post migration factors as the predictor variables across the five years of follow-up.

**Table 3:** Final model results from multivariate logistic regression analysis using chronic pain and long-term disability as the outcome. OR-odds ratio; *p* = *p*-value; CI = confidence interval

Variable	Response	Chronic Pain			Long-term Disability		
		***OR***	***P***	***95% CI***	***OR***	***P***	***95% CI***
***PRE-MIGRATION FACTORS***							
**Age**	18 – 75 years	1.07	< 0.001	(1.04 – 1.09)	1.03	0.060	(1.00 – 1.06)
**Visa Category**	200 Refugee Visa	-	-	-	-	-	-
	Onshore Protection/ Humanitarian Visa	1.22	0.678	(0.47 – 3.16)	1.08	0.895	(0.36 – 3.22)
	204 Women at Risk Visa	2.25	0.012	(1.20 – 4.24)	1.70	0.249	(0.69 – 4.13)
***POST-MIGRATION FACTORS***							
**General Health**	Poor – Very poor	-	-	-	-	-	-
	Good – Fair	0.17	< 0.001	(0.08 – 0.35)	0.16	< 0.001	(0.07 – 0.39)
	Excellent – Very Good	0.06	< 0.001	(0.02 – 0.16)	0.04	< 0.001	(0.01 – 0.14)
**Region of Settlement in Australia**	Regional Australia	-	-	-	-	-	-
	Metropolitan cities	0.44	0.052	(0.19 – 1.01)	0.47	0.178	(0.16 – 1.41)
**Stress – Discrimination**	No	-	-	-	-	-	-
	Yes	13.78	< 0.001	(3.15 – 60.21)	1	-	-

#### Association between pre-and post-migration factors in women who reported both chronic pain and long-term disability

Given that people with chronic pain are likely to have a long-term disability [29], a sub-group analysis was undertaken to examine the factors associated with chronic pain in women who also reported having a long-term disability, injury of health condition. Of the 310 women, 45% (*n* = 139), reported having chronic pain, and among these women a further 66% (*n* = 120), also reported having a long-term disability or health condition that had lasted 12 months or more.

Pre-migration factors included in the multivariate analysis for chronic pain and long term-disability from univariate analysis were age, number of pre-migration traumas, marital status, education and visa class (Table 2). After controlling for confounding factors, age remained a significant pre-migration factor associated with chronic pain in women who also reported having a long-term disability (OR 1.01; 95% CI 1.01, 1.08). Interestingly, women who report experiencing 1–2 traumas before migrating to Australia had 3.3 times the odds of having chronic pain and a long-term disability than women who report no history of trauma (OR 3.30; 95% CI 1.05, 10.34).

Post-migration factors included in the multivariate analysis for chronic pain and long term-disability were main income source, number of financial hardships, financial stressors, English language proficiency, language barrier, PTSD, general health, region of settlement in Australia, loneliness and discrimination. The selection of these variables was based on previous univariate analyses. The results of this multivariate regression analysis between post migration factors, chronic pain and long-term disability can be found in Table 2. Findings indicate that general health was significantly associated with chronic pain in women who also reported a long-term disability (OR 0.04; 95% CI 0.01, 0.16).

In our final model (Table 3), the sub-group analysis demonstrated that general health remained significantly associated with chronic pain in women who reported concomitant long-term disability. Women who reported “Excellent – very good” general health had 96% less odds of having chronic pain (OR 0.04; 95% CI 0.01, 0.14) while those who reported “Good – fair” had 84% less odds of reporting chronic pain (OR 0.16; 95% CI 0.07, 0.39).

## Discussion

This study has shown that pre-migration factors, but more importantly post-migration factors, are associated with self-reported chronic pain and long-term disability in refugee women. Post migration experiences such as general health, discrimination and region of settlement were most likely to be associated with chronic pain.

In line with existing research showing a significant association between chronic pain and general health [30], our study also found that refugee women who reported poorer general health had greater odds of self-reporting chronic pain. Interestingly, the rates of poorer general health also appear to be greater in the initial years of resettlement in Australia [31]. This suggests that there may be many unmet health needs that are compounded by the challenges of resettlement, highlighting the need for greater clinical awareness to help inform and prepare refugee health care and settlement service providers to improve chronic pain management for refugee women who have been systematically marginalised. However, the complexity of health care provision is also influenced by the location refugee women resettle into. Our study has shown that refugee women who resettled in more rural or remote parts of Australia had greater odds of experiencing chronic pain and our interaction effects demonstrated that general health was also influenced by settlement location. Geographic isolation and the scarcity of specialized services affects the experiences of immigrant and refugee women in regional/remote areas [32] meaning that acute conditions such as pain are also more likely to persist longer than needed. Therefore, we need to consider specific strategies that resettlement services, clinicians and policy makers can implement to mitigate the negative effects of migration on chronic pain in refugee women during their initial years of resettlement in Australia.

It has been extensively reported that Australians residing in rural or regional parts of the country have shorter lives, higher levels of disease and injury and poorer access to health care services compared to people living in more metropolitan regions of the country [33, 34]. Meanwhile, primary and allied health services are extremely limited in rural and regional Australia making access to best practice pain management more difficult [34]. A study by Sypek and colleagues found that the difficulties faced by rural Australians securing equitable access to health services are considerably amplified for refugees [35]. Providing opportunities that are available through rural centres or telehealth where women can readily access resources for English upskilling, multicultural supports and health services, including mental health support is needed but would require a strategy to inform women that these opportunities exist and that they are easily accessible. Furthermore, there is a fragility in the health services offered in rural regions to provide a comprehensive approach to chronic pain care. This is in part, a result of a low number of practitioners, high turnover of staff, resulting in an attrition of specialised knowledge among health care workers treating refugees [35]. However, it is not clear how the barriers to access chronic pain management services in rural and remote regions for refugee women may differ from the broader population. Future research in this field could be enhanced by understanding community perspectives of refugee women living in rural and regional parts of Australia who experience chronic pain. These perspectives can guide new and emerging communities’ social integration, sense of belonging and chronic pain settlement outcomes for refugee women.

The post-migration factor that was most significantly associated with reporting chronic pain in our study was stress arising from discrimination. Experiences of discrimination are commonly reported in the resettlement accounts of refugees in Australia and feature prominently in settings of social support, in work and within neighbourhoods [36–40]. A recent study showed that 22% of refugees who resettled in South Australia reported experiencing discrimination, the majority of whom felt that the discrimination had negatively affected their health [41]. A national survey in the United States also found that 4.1 million Americans who experienced chronic pain reported that it was caused by an increase in psychological distress arising from perceived discrimination. Colleagues Fozdar and Torezani suggest that some refugees, particularly women, may consider discrimination to be a single, or rather an individual phenomenon, as opposed to a system that is fundamentally intended to disadvantage them, and therefore feel it is less damaging [36]. Interestingly, previous research has shown that refugee women may buffer the negative impacts of stigmatisation on their health by responding to accounts of discrimination through various cognitive, affective and behavioural pathways [42]. However, there is limited research that directly examines the association between discrimination and chronic pain in women from a refugee background [18, 38, 40, 43–48]. Greater insight into how refugee women respond to discrimination may help to understand and possibly interrupt the pathways with which stress from discrimination may impact chronic pain [42]. However, it is important to note that discrimination is a consequence of broader systemic issues and policies that seek to mitigate stigmatisation should not place the burden of responsibility on those who experience discrimination [47].

Consistent with previous findings, pre-migration factors such as age was a risk factor for chronic pain in our cohort of refugee women [49]. Interestingly, our study also showed that migration pathway may also be an important pre-migration predictor of chronic pain. Women who migrated under the 204 Women at Risk visa category had greater odds of reporting chronic pain than women who migrated under any other visa subclass. This may be because women who arrive under the Women at Risk visa category are especially vulnerable to gender-related human rights violations in addition to sufferings often reported by other refugee groups [50]. As a result, their experience and plight for refuge will likely differ from that of their male or not at-risk female counterparts. This finding extends on previous research that shows how recently resettled refugee women living in Australia who are at risk have substantially greater odds of experiencing psychiatric distress such as anxiety, post-traumatic stress disorder, depression in addition to considerable symptom somatization [51]. Likewise, Schweitzer and colleagues revealed that the proportion of women-at-risk who had been exposed to serious injury, detention or imprisonment, were missing or kidnapped, or experienced rape or sexual abuse was over double that reported by women who resettled through other humanitarian and refugee visa pathways [52, 53]. The unique and difficult events described by refugee women-at-risk suggests that many of them will likely experience longer term effects to their health in the post-migration setting. Developing an understanding of the factors that significantly impact chronic pain in resettled women-at-risk benefits health practices and holds particular worth in guiding resettlement policies and programs for individuals who are systematically marginalised.

In our cohort of refugee women, approximately two-thirds of the women who reported chronic pain also experienced a long-term disability, injury or health condition. This suggests that chronic pain may impede their ability to function day to day, or integrate into their community and may subsequently result in further isolation [47, 54]. However, the percentage of women who had a disability identified at arrival was 37%, whilst five years into resettlement the percentage of women who identified as having a disability dropped to 13%. This ‘change’ over five years may be due to a number of post-settlement factors, however, due to limitations in our dataset it is unclear why this decrease occurred. Furthermore, the severity of tissue injury does not appear to be a good indicator or predictor of eventual disability. Jamison and colleagues found that psychological factors play the most prominent role in whether someone has a pain related disability after an injury [55]. This raises an interesting approach to clinical care for chronic pain sufferers, and more specifically refugee women. Future research in this field could be enhanced by considering better initial pain control which could have an effect of reducing long-term disability and may also work to break fear-avoidance patterns and pessimism around pain [55].

Chronic pain care is complex and oftentimes overwhelming for both the patient and clinician. A study by Bifulco and colleagues demonstrated that chronic pain is underdiagnosed and undertreated in primary care and this is likely to be even greater for people who are systemically marginalised [56]. With a burgeoning number of refugee women reporting chronic pain, a new strategy that values the complex nature of both chronic pain and refugee health concerns is needed to adequately meet these challenges in clinical practice. Mainstream health pathways such as primary care may benefit from the implementation of large, multisite screening tools that evaluates both pain and function upon arrival [56]. Whilst the current study doesn’t support this finding, it does suggest that further research may benefit from exploring refugee women’s perceptions of pain to help develop a guide that considers both pain and function in a routine screening process for health professionals who care longer-term for women belonging to refugee-like backgrounds [56]. To achieve this, a coordinated long term national and state funded approach to ensure consistency of such services to these women is needed. A central or state government funded body such as post-settlement contractors who work in partnership with existing refugee health services and primary care to deliver resources and time for women belonging to refugee-like backgrounds are in an essential position to assist. Resettlement contractors should be funded and supported sufficiently to provide continuity of care for new arrivals over the first five years, rather than short term tenders. A national body that offers a continued record may not only enhance the health and wellbeing of refugee women but overtime allow for further follow up strategies to help positively impact the perceptions and management of their chronic pain.

This study has considered the impact of migration on refugee women’s reporting of chronic pain in Australia. Nevertheless, the results of this study need to be considered in the context of the research limitations. This was a secondary analysis of the BNLA study which did not have the current assumption in mind when the project was first initiated. We were therefore limited to available study variables and we acknowledge that factors such as pre-departure/transit experience, family domestic violence pre- and post-resettlement trauma that may be associated with chronic pain in this population were not captured and included in our analyses. Additionally, there was no specific chronic pain question, nor was our outcome variable measured at every time point. The sampling of women was also restricted to primary applicants, as secondary applicants were not asked the comprehensive version of the survey which omitted the primary outcome variable and several covariates, reducing our sample size. This also meant that the majority of women in our cohort reported good English proficiency, which differs considerably when comparing to other national resettlement trends where humanitarian entrants, particularly women, are reported to have low levels of English proficiency and formal education suggesting that our sample size is small and non-representative of most humanitarian entrant women. Furthermore, the sample also does not reflect asylum seeker cohorts as only 0.7% experienced detention which limits our ability to translate our findings more broadly to those from refugee-like backgrounds.

The capacity for respondents to use the self-administered or interview-administered formats of the assessment may have also introduced some inconsistencies [57]. The measurements were based on self-report questionnaires; therefore, responses are susceptible to bias (e.g., recall bias and social desirability) [57].Future research may benefit from standardised measurement instruments to improve the reliability of data reported and enhance comparability across studies. Our study did not include potential moderating factors such as 1) access to a GP, (2) postcodes (and relative ISRAD/IRSD deciles), (3) linkages to community or multicultural groups, (4) access to extended family within Australia, (5) literacy in primary language, (6) median number of children/dependents, and recommend that future studies incorporate these to provide an even greater level of understanding of the context that these women are in. Finally, causal associations cannot be defined because of the nature with which we defined our chronic pain variable omitted the temporality of pain over time. Studies using prospective designs are needed to determine the causal effects of pre-migration and post-migration factors on refugee women’s chronic pain health.

## Conclusion

These limitations notwithstanding, this study provides new and emerging evidence that both pre- and post-migration factors such as age, migration pathway, region of settlement, discrimination and general health are associated with chronic pain in refugee women. Interestingly, we found that post migration factors had greater odds of predicting chronic pain than pre-migration factors, suggesting that the post-migration environment is integral for the adequate management of chronic pain and highlights the importance of settlement support services as a key moderator for long-term health outcomes. In addition to this, long-term disability was exhibited by the majority of women who reported chronic pain, compounding the challenges refugee women may face when trying to access post-settlement health services or social support. In light of these findings, clinicians, policy makers and health care providers should consider the opportunities available to reduce the stressors experienced in the post-migration setting, and implement practices that may minimise refugee women’s ongoing stressors. Involvement of social integration services that assist refugee women’s ability to engage with the health services they need is an important consideration for improving the chronic pain outcomes of refugee women who are systematically marginalised in Australia and other countries of resettlement.

## Supplementary Information

**Additional file 1.**

## Data Availability

The de-identified BNLA data was obtained (with approval) from the Australian Government’s Department of Social Services. All datasets used in this study are publically available from the Australian Government’s Department of Social Services at the website: https://www.dss.gov.au/national-centre-for-longitudinal-data-ncld/access-to-dss-longitudinal-datasets.

## Abbreviations

AIC: Akaike’s Information Criterion
AIFS: Australian Institute of Family Studies
BNLA: Building a New Life in Australia
NHMRC: National Health and Medical Research Council
PA: Primary applicant
VIF: Variance Inflation Factor
