# Supplementary material for: The association between chronic pain and pre-and-post migration experiences in resettled humanitarian refugee women residing in Australia
Source: BMC Public Health. 2022 May 7;22:911. doi: 10.1186/s12889-022-13226-5 (PMC9080158; doi:10.1186/s12889-022-13226-5)
Supplement: Supplementary file 1 — Additional file 1. [file 12889_2022_13226_MOESM1_ESM.docx]

Univariate Analysis - Pre-and Post-Migration Factors

| **Variable** | **Response** | | **Chronic Pain** | | |
| --- | --- | --- | --- | --- | --- |
|  |  |  | ***OR*** | ***P*** | ***95% CI*** |
| ***PRE-MIGRATION FACTORS*** | | | | | |
| **Age** | 18 – 75 years | | 1.08 | 0.000 | (1.06 – 1.11) |
| **No. of pre-migration traumas experienced** | None | | - | - | - |
|  | 1 – 2 | | 1.87 | 0.110 | (0.87 – 4.01) |
|  | 3 or more | | 2.81 | 0.011 | (1.26 – 6.26) |
| **Married or has a partner** | No | | - | - | - |
|  | Yes | | 0.45 | 0.002 | (0.27 – 0.74) |
| **Pre-arrival Education** | Never attended school | | - | - | - |
|  | < 6 years of school | | 0.69 | 0.261 | (0.36 – 1.32) |
|  | 6 – 12 years of school | | 0.42 | 0.009 | (0.22 – 0.81) |
|  | 12 years + of school | | 0.40 | 0.013 | (0.19 – 0.82) |
|  | Trade or Tech school | | 0.81 | 0.708 | (0.27 – 2.45) |
|  | University Degree | | 0.24 | 0.003 | (0.09 – 0.62) |
| **Visa Category** | 200 Refugee Visa | | - | - | - |
|  | Onshore Protection/ Humanitarian Visa | | 1.37 | 0.414 | (0.65 – 2.90) |
|  | 204 Women at Risk Visa | | 2.30 | 0.001 | (1.41 – 3.73) |
| ***POST-MIGRATION FACTORS*** | | | | | |
| **Main income source in Australia** | Non-government support | - | | - | - |
|  | Government support | 6.11 | | 0.018 | (1.36 – 27.35) |
| **No. daily financial hardships** | None | - | | - | - |
|  | 1 or more | 1.46 | | 0.102 | (0.93 – 2.29) |
| **Financial stressors** | No | - | | - | - |
|  | Yes | 1.82 | | 0.010 | (1.15 – 2.88) |
| **English speaking proficiency** | not well/not at all | - | | - | - |
|  | very well/well | 0.47 | | 0.013 | (0.26 – 0.85) |
| **Stress- language barriers** | No | - | | - | - |
|  | Yes | 2.40 | | 0.001 | (1.44 – 3.98) |
| **Mental Health Status PTSD8** | No | - | | - | - |
|  | Yes | 2.46 | | 0.000 | (1.54 – 3.92) |
| **General Health** | Poor – Very Poor | - | | - | - |
|  | Excellent – Very Good | 0.19 | | 0.000 | (0.12 – 0.30) |
| **Region of settlement in Australia** | Regional Australia | - | | - | - |
|  | Metropolitan cities | 0.55 | | 0.061 | (0.30 – 1.03) |
| **Stress-loneliness** | No | - | | - | - |
|  | Yes | 2.18 | | 0.004 | (1.28 – 3.71) |
| **Discrimination** | No | - | | - | - |
|  | Yes | 13.18 | | 0.015 | (1.67 – 104.26) |

Collinearity – Pre-and Post-Migration Factors

| Variable | VIF | Tolerance = I/VIF |
| --- | --- | --- |
| *PRE-MIGRATION FACTORS* |  |  |
| Age  Number of Pre-migration Traumas  Married or Partnered  Pre-arrival Education  Visa Category | 1.10  1.04  1.34  1.07  1.34 | 0.912  0.964  0.748  0.935  0.748 |
| *POST-MIGRATION FACTORS* |  |  |
| Main income source in Australia  No. daily financial hardships  Financial stressors  English speaking proficiency  Stress- language barriers  Mental Health Status PTSD8  General Health  Region of settlement in Australia  Stress-loneliness  Discrimination | 1.05  1.17  1.28  1.33  1.37  1.19  1.21  1.11  1.16  1.06 | 0.950  0.855  0.781  0.754  0.729  0.838  0.825  0.900  0.861  0.948 |
